# Supplementary material for: The Effects of the Follicle-Stimulating Hormone on Human Follicular Fluid-Derived Stromal Cells
Source: Int J Mol Sci. 2023 Jan 26;24(3):2450. doi: 10.3390/ijms24032450 (PMC9916742; doi:10.3390/ijms24032450)
Supplement: Supplementary file 1 [file ijms-24-02450-s001.zip › Supplementary table S1.pdf]

Supplementary Table S1. Antibodies used in flow cytometry analysis

**Antibody**	**Fluorophore**	**Isotype**	**Cat. No.**
CD90	APC	IgG1 κ	1A-652-T100, exbio
CD44	APC	IgG1, κ	338805, biolegend
HLA-ABC	488	IgG2a, κ	311413, biolegend
CD166	PE	IgG1, κ	343903, biolegend
CD73	FITC	IgG1, κ	1F-675-T100, exbio
CD13	APC	IgG1	1A-396-T100, exbio
CD105	APC	IgG2a	1A-298-T100, exbio
CD56	APC	IgG1, κ	362504, biolegend
CD146	488	IgG1, κ	361020, biolegend
BMSC	APC	IgG1, κ	106712, biolegend
CD9	FITC	IgG1	1F-208-T100, exbio
SUSD2	APC	IgG1	1A-835-C100, exbio
CD338	APC	IgG2b, κ	332020, biolegend
CD34	FITC	IgG2a, κ	343604, biolegend
CD45	FITC	IgG1, κ	982316, biolegend
CD140b	APC	IgG1	1A-590-T100, exbio
